# Associations Between Breast Implant Characteristics, Autoimmune Disease History, and Post-augmentation Symptom Reporting Among Puerto Rican Women

**DOI:** 10.7759/cureus.89698

**Published:** 2025-08-09

**Authors:** Sergio M Aymat Sánchez-Vahamonde, Maillim I Ortiz López, Samuel Padua, Barbara Riestra

**Affiliations:** 1 School of Medicine, Universidad Central del Caribe, Bayamon, PRI; 2 Department of Interventional Radiology, Manatí Medical Center, Manati, PRI

**Keywords:** breast implant, breast implant complications, post-implant symptoms, puerto rico, saline implant, textured breast implant, textured implants

## Abstract

Background

Breast augmentation surgery (BAS) is one of the top cosmetic surgical procedures performed in the United States every year. There are various breast implant options, such as saline, silicone, smooth, and textured implants. Breast implant illness (BII) is a disorder associated with a wide array of symptoms presenting post breast implant surgery and is often associated with autoimmune disorders. Despite its prevalence, no comprehensive studies have investigated the occurrence of concerning symptoms after breast implant surgery in Puerto Rico (PR), and specific risk factors remain unidentified. This study aims to address this knowledge gap, evaluating self-reported symptom presentation post BAS in Puerto Rican women with respect to their specific implant type, surface finish, and past medical history.

Methods

This was a cross-sectional, quantitative survey study. A structured 18-item questionnaire was administered to Puerto Rican women over the age of 21 who had undergone breast augmentation. Participants were recruited through social media, referrals, and a breast radiology clinic. The survey included questions on demographics, medical history, implant characteristics, and symptomatology. Descriptive and inferential statistical analyses were performed.

Results

Among the 361 complete survey responses evaluated, 56.7% of participants reported having silicone implants, 35.8% saline, and 5.2% were unsure of their implant type. Additionally, 11.3% of participants had textured implants, 52.1% smooth, and 36.4% were unsure of their implant's surface finish. Overall, 30.6% of participants reported experiencing concerning symptoms following implant placement. The most prevalent symptoms were anxiety or depression (69.4%), body or joint pain (66.7%), cognitive or memory dysfunction (63.9%), muscle pain (54.6%), and abnormal fatigue (50%). When evaluating symptoms based on implant surface finish, 41.02% of those with textured implants reported concerning symptoms, compared to 27.02% of those with smooth implants. However, a chi-square test did not find a significant association between implant type and symptom presentation (p=0.250) or between surface finish and symptom presentation (p=0.085). Personal history of autoimmune disease was significantly associated with increased symptom reporting (odds ratio (OR) = 2.77; 95% CI: 1.67-4.59), whereas family history was not. Symptom prevalence was highest among individuals aged ≥55 (44.4%) and those with implants placed five to nine years prior (39.7%).

Conclusion

Patients in PR experienced concerning symptoms following breast implant surgery. Frequently reported symptoms such as anxiety, depression, body/joint pain, and cognitive dysfunction may suggest a possible link to mental health. No significant association was found between the type of implant and the development of these symptoms. This finding suggests that other factors, such as individual patient susceptibility or environmental influences, may play a critical role in symptom development. However, there was a trend indicating that patients with textured implants and those with a history of autoimmune disease may experience symptoms at a higher rate. This trend underscores the need for further research aimed at collecting more comprehensive data, including longitudinal follow-up, to better understand the potential mechanisms behind these symptoms and to identify specific risk factors within the Puerto Rican population.

## Introduction

Breast augmentation surgery (BAS) ranks among the most frequently performed cosmetic procedures in the United States and Puerto Rico (PR), representing a significant aspect of modern aesthetic practice [1]. The procedure’s popularity is driven not only by aesthetic enhancement but also by evolving techniques and improvements in implant technology. Today, a wide range of implant options is available, from saline and silicone fillers to varied surface finishes such as smooth and textured. Each offers distinct physical characteristics and biological interactions [2].

Despite the prevalence of BAS, the decision-making process for both patients and surgeons remains complex. Implant selection involves a careful consideration of factors including biocompatibility, longevity, and potential postoperative complications. For instance, saline implants are valued for their safety in rupture scenarios, as the saline solution is naturally absorbed and excreted by the body; however, they may not offer the natural feel provided by silicone implants, which may require periodic monitoring due to potential silent ruptures [2,3]. Furthermore, the exterior finish of an implant can influence tissue response, with textured implants designed to reduce movement by encouraging tissue adherence, yet potentially increasing the risk for adverse inflammatory or immune-mediated reactions [4].

In recent years, news articles and studies published on breast implant illness (BII) have generated considerable discussion regarding systemic symptoms reported by patients following augmentation [5]. The literature predominantly addresses BII as a broad collection of symptoms without differentiating between the various implant characteristics and without demonstrating a causal relationship between implants and the appearance of these symptoms [6,7]. It is important to note that systematic reviews performed in 2000, 2007, and 2016 have not found any association between breast implants and any type of connective tissue disease, rheumatic disorders, or other self-reported symptoms [6,8,9]. However, women across the country have continued to report concerning symptoms after having breast implant surgery, and there is a lack of studies addressing these patients. This is particularly true in the context of Puerto Rican populations, where very limited research exists on how specific implant features, such as filler type and surface finish, may contribute to the onset of localized and systemic symptoms.

Recent systematic reviews have shown that individuals with breast implants are significantly more likely to report systemic symptoms such as fatigue, myalgia, joint pain, and cognitive difficulties compared to controls [10]. A large international cohort study further described severe symptomology, including fatigue and arthralgia, in a sample of 275 women with implants, many of whom had consulted multiple medical specialties for their symptoms [11]. These findings underscore the need to assess how these patterns manifest in Puerto Rican women with implant devices.

This study shifts focus from the generalized categorization of BII to a detailed evaluation of symptom patterns that arise in Puerto Rican women after their BAS. The objective of this study is to determine the prevalence of self-reported post-augmentation symptoms and investigate their associations with key predictors, including implant type, surface finish, and personal or family history of autoimmune disease, in a cohort of Puerto Rican women. In doing so, our research aims to bridge a critical knowledge gap by examining whether differences in implant type and surface finish are associated with distinct symptom profiles. By integrating demographic, clinical, and surgical data with robust statistical analyses, this study seeks to enhance clinical decision-making, refine patient counseling strategies, and stimulate further research into the long-term outcomes of breast augmentation procedures [12,13]. In essence, understanding these nuances will empower both physicians and patients to make informed choices tailored to the unique characteristics of the Puerto Rican population.

## Materials and methods

Study design and population

This was a quantitative, cross-sectional study conducted among Puerto Rican women aged 21 years or older who had undergone BAS. Participants were recruited using social media outreach, referrals, and at a breast radiology center in Manatí, PR. Eligibility required residency in PR at the time of the survey.

Survey instrument

Data were collected using an anonymous 18-item survey developed by the research team. It assessed four domains: (1) demographic characteristics; (2) personal and family medical history (including autoimmune conditions and allergies); (3) implant variables (type and surface finish); and (4) post-augmentation symptoms. Symptoms included both physical (e.g., fatigue, joint pain) and psychological (e.g., anxiety, cognitive dysfunction) domains. It consisted of fixed-response (yes/no, multiple choice) questions. This instrument was not formally psychometrically validated but was reviewed by subject-matter experts and pilot-tested to ensure clarity and relevance (Appendix A).

Ethical approval

The study received Institutional Review Board (IRB) approval (IRB No. 2023-17) from Universidad Central del Caribe. Bayamon, PR (approval number: 2023-17). All responses were anonymous, and informed consent was obtained prior to participation.

Statistical analysis

Data were analyzed using both descriptive and inferential statistics. Symptom prevalence was summarized using means and proportions. Group comparisons between implant types and surface finishes were conducted using independent sample t-tests and chi-square tests, respectively. Additionally, regression models were applied to evaluate potential associations between implant characteristics and symptom burden. Participants who selected “unknown” in response to implant type or surface finish were retained in the overall descriptive analyses to provide a complete representation of the study population. However, these responses were excluded from subgroup inferential analyses, including chi-square tests and logistic regression, to avoid introducing bias or misclassification into comparisons between defined implant categories. This approach ensured the integrity of statistical testing while maintaining transparency about the full dataset. Statistical significance was defined as p < 0.05.

## Results

Demographics and implant characteristics

**Figure 1:**
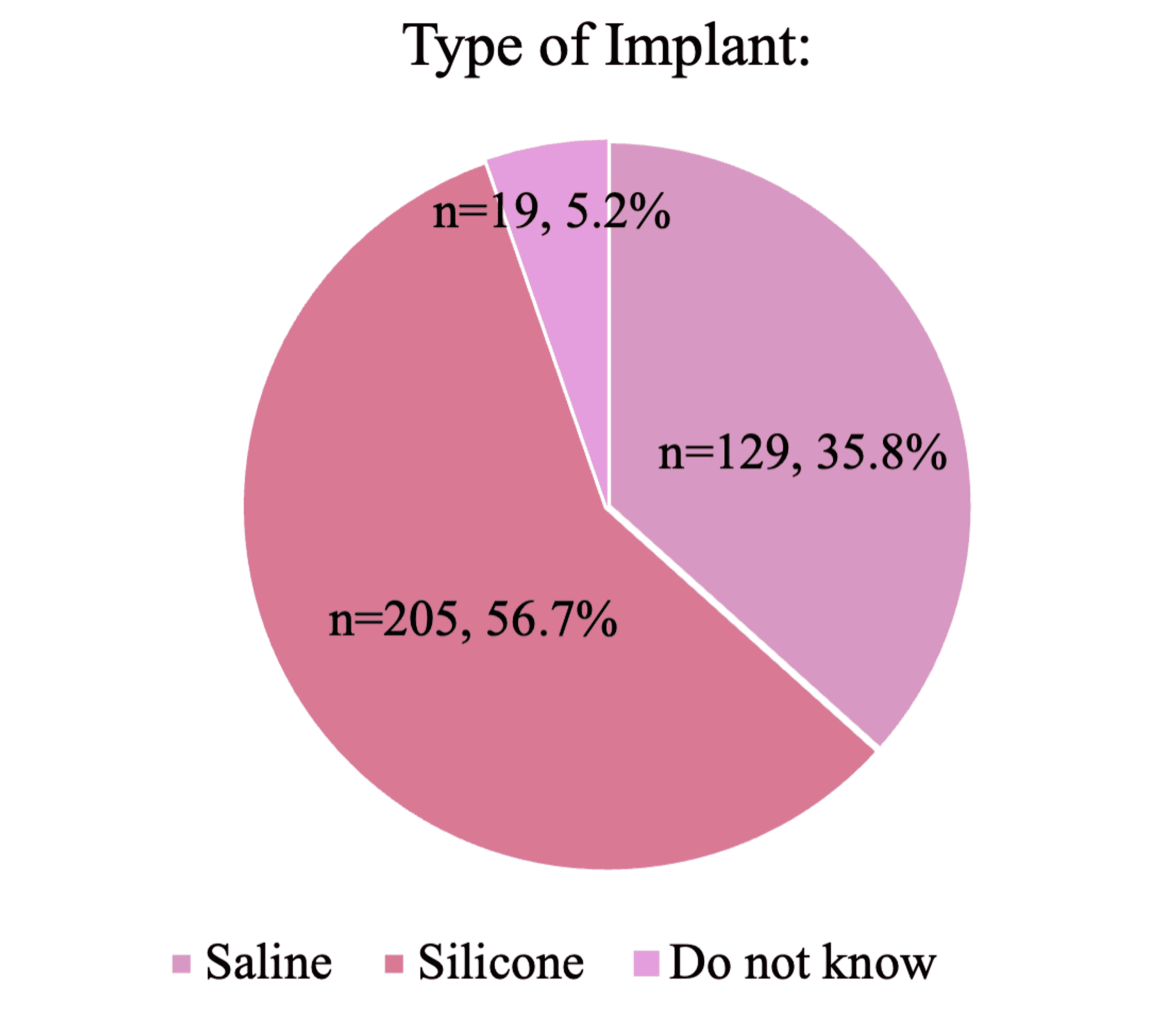
The pie chart shows the distribution of implant filler types (silicone, saline, unknown) among respondents. Data are presented as % and N; N=361

**Figure 2:**
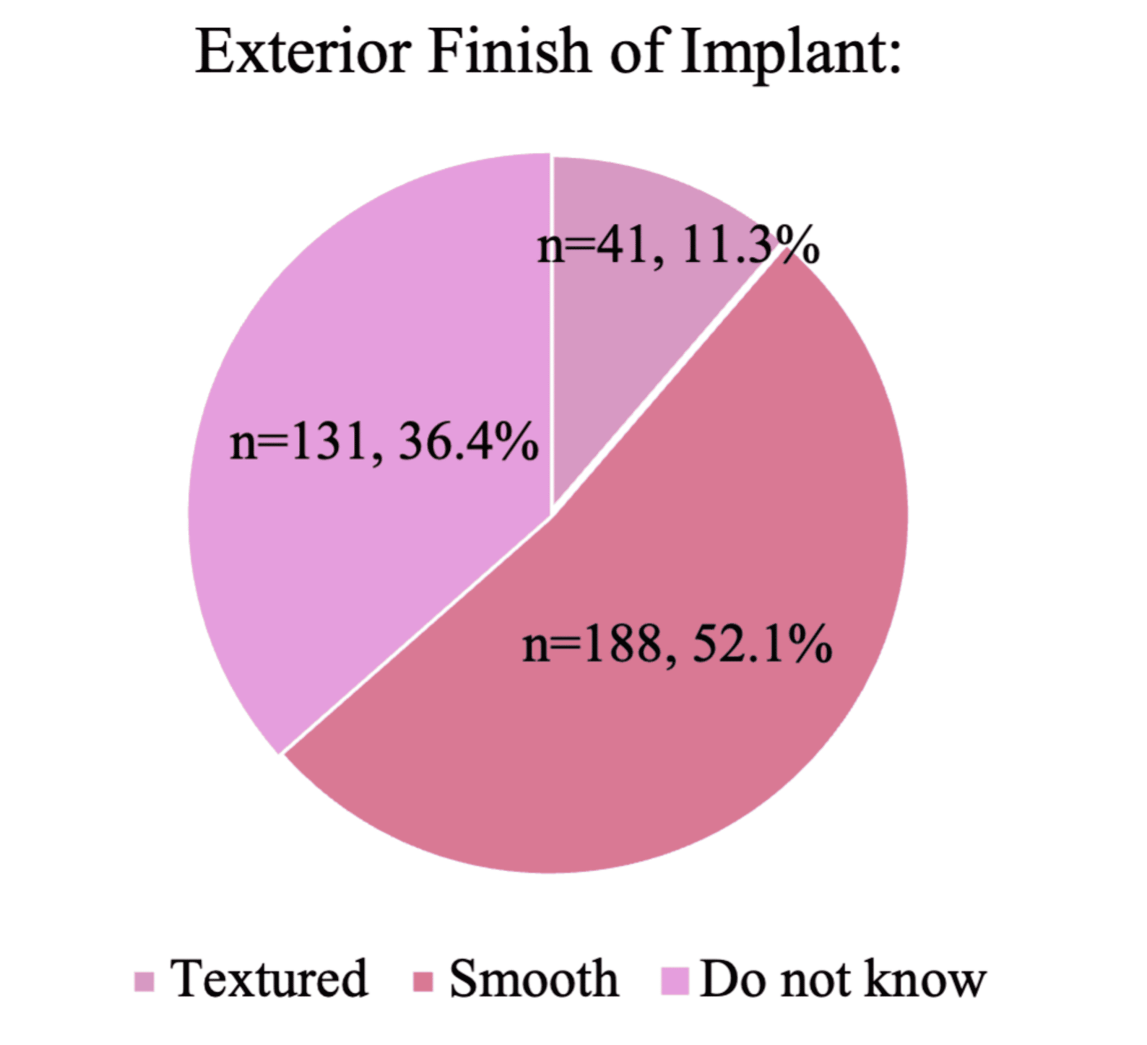
The pie chart shows the distribution of the implant's exterior surface finish (smooth, textured, unknown) among respondents. Data are presented as % and N; N=361

Among the 361 survey respondents, 56.7% (n = 205) indicated having silicone implants, 35.8% (n = 129) saline implants, and 5.2% (n = 19) were uncertain of their implant type (Figure 1). Furthermore, 11.3% (n = 41) reported having textured implants, 52.1% (n = 188) smooth implants, and 36.4% (n = 131) were not sure of the surface finish of their implants (Figure 2).

Symptom prevalence

A total of 30.6% of participants reported experiencing symptoms they attributed to their breast implants. The most commonly endorsed symptoms were anxiety and/or depression (69.4%, n=77), body or joint pain (66.7%, n=73), cognitive or memory dysfunction (63.9%, n=70), muscle pain (54.6%, n=60), and abnormal fatigue (50.0%, n=55).

Statistical associations with implant type and surface

Analysis of symptom prevalence by implant type revealed no statistically significant difference between respondents with silicone implants and those with saline implants (p = 0.250). While not reaching statistical significance, a trend was observed in relation to implant surface finish: 41.0% (n=16) of respondents with textured implants reported symptoms compared to 27.0% (n=51) of those with smooth implants (p = 0.085) (Table 1).

**Table 1:** Association Between implant characteristics and symptom reporting Data presented as N (%); statistical test: Chi-square test of independence; significance level set at p < 0.05.

Variable	Symptom reporting n (%)	No symptoms n (%)	Test statistic (χ²)	p-value
Implant type			1.32	0.25
Silicone (N = 205)	68 (33.2%)	137 (66.8%)		
Saline (N = 129)	35 (27.1%)	94 (72.9%)		
Surface finish			2.96	0.085
Textured (N = 41)	16 (41.0%)	25 (59.0%)		
Smooth (N = 188)	51 (27.0%)	137 (73.0%)		

Autoimmune and cancer history

A logistic regression model was employed to assess associations between symptom reporting and medical history variables. Participants with a personal diagnosis of an autoimmune condition had significantly greater odds of reporting symptoms compared to those without such a diagnosis (odds ratio (OR) = 2.77; 95% confidence interval (CI): 1.67-4.59). A positive family history of autoimmune or connective tissue disease showed a non-significant trend toward increased symptom reporting (OR = 1.51; 95% CI: 0.90-2.53) (Table 2).

**Table 2:** Logistic regression analysis of autoimmune history and symptom reporting OR: odds ratio; CI: confidence interval; statistical test: logistic regression using Wald Chi-square test; significance level set at p < 0.05.

Predictor	Odds ratio (OR)	95% Confidence interval (CI)	Test statistic (Wald χ²)	p-value
Personal autoimmune diagnosis	2.77	1.67 – 4.59	17.89	<0.001
Family history of autoimmune conditions	1.51	0.90 – 2.53	2.56	0.109

Stratified analyses by age and time since implantation

Symptom prevalence varied across age groups. Among respondents aged 55 years and older, 44.4% (n=12) reported experiencing symptoms, compared to 30.4% (n=31) in the 45-54 years age group, 28.5% (n=37) in the 35-44 years age group, and 28.5% (n=20) in those under 35 years. Stratification by time since implantation revealed the highest symptom prevalence in individuals whose implants had been placed five to nine years prior (39.7%, n=27), followed by those with implants for 10 or more years (27.9%, n=43). The lowest prevalence was observed in participants with implants placed within the last five years (22.0%, n=25). These patterns suggest a possible increase in symptom burden with age and a peak in symptom reporting in the mid-term post-implantation period.

## Discussion

The findings of this study demonstrate no significant association between breast implant filler type, silicone versus saline, and the development of post-augmentation symptoms within the surveyed population. However, a trend toward higher symptom prevalence was observed among individuals with textured implants, consistent with prior hypotheses suggesting that textured surfaces may provoke more substantial inflammatory or immunologic responses due to increased surface area and tissue integration [4].

Emerging evidence supports the notion that breast implants, particularly textured types, can elicit a range of systemic symptoms collectively referred to as BII. Studies have suggested that biofilm formation on implant surfaces may trigger chronic immune activation, contributing to the development of symptoms [14]. Additionally, immunologic studies have noted increased levels of pro-inflammatory cytokines and immune dysregulation in symptomatic patients, lending further biological plausibility to patient-reported symptoms [15].

A salient finding in this cohort was the significantly higher likelihood of symptom reporting among individuals with a personal history of autoimmune disease, a result in alignment with previous large-scale analyses. For example, a population-based study by Magno-Padron et al. reported a high prevalence of autoimmune symptoms among breast implant recipients, further reinforcing concerns about the immunogenicity of implant materials [16]. More recently, a systematic review by Rohrich et al. emphasized the need for careful patient selection and preoperative counseling regarding potential long-term immunological outcomes [17].

Additionally, the stratified analysis revealed an age-related increase in symptom prevalence, with the highest rates observed among patients aged ≥55 years and those who had undergone implantation five to nine years prior. These findings align with the model of chronic exposure leading to cumulative immune system activation and symptom development over time, as supported by prospective cohort studies showing symptom improvement following explantation [18].

Nonetheless, the etiology of BII remains complex and multifactorial. While immunologic theories predominate, psychological and somatic factors are increasingly recognized as important contributors to symptomatology [19]. Importantly, recent efforts have highlighted the heterogeneity of BII presentations and called for the development of standardized diagnostic criteria and patient-reported outcome measures to improve research comparability and clinical management [20].

Limitations

This study is subject to several limitations. First, although the sample size is sufficient for exploratory analysis, it may lack the statistical power to detect more subtle associations. Second, the reliance on self-reported data introduces both recall and reporting bias. This is particularly relevant given that 36.4% of participants were unsure of their implant’s surface finish, highlighting the potential for misclassification and subjective reporting. Third, the cross-sectional design and lack of a control group limit the ability to infer causality between implant characteristics and reported symptoms. Finally, recruitment through social media and a radiology center may have introduced selection bias, as individuals experiencing symptoms or actively seeking validation for suspected BII may have been more likely to participate. 

## Conclusions

This study offers a preliminary examination of symptomatology presenting after BAS among Puerto Rican women and highlights potential associations with implant characteristics and patient history. Although no statistically significant associations were identified with implant filler type or surface texture, the observed trends, particularly the elevated symptom reporting associated with textured implants and the statistically significant association identified with autoimmune disease, warrant further investigation. Future research involving larger, prospectively followed cohorts will be essential to validate these findings, clarify etiologic mechanisms, and guide evidence-based clinical decision-making to optimize patient safety and outcomes.

## Appendices

Appendix A 

**Table 3:** Post-breast augmentation symptom presentation: survey questionnaire This questionnaire was used to collect data on breast implant characteristics, symptomatology, and medical history among participants. Responses were collected anonymously.

Question	Response options
Date of birth	Open-ended (e.g., January 7, 1989); must be over 21 years of age to participate
What is your ethnicity?	Check all that apply: American Indian or Alaska Native, Asian, Black or African American, Native Hawaiian or Other Pacific Islander, White, Other (Specify), I prefer not to answer
Have you undergone breast implant surgery?	Yes, No
For what reason were the implants placed?	Reconstruction (Due to cancer), Reconstruction (Due to Asymmetry), Augmentation, I prefer not to answer
Date the implants were placed	Open-ended (e.g., January 7, 2019)
Type of implant	Saline, Silicone, Both, Don’t know, I prefer not to answer
Outside finish of the implant	Textured, Smooth, Don’t know, I prefer not to answer
Have you experienced any troubling symptoms that have impacted your daily activities after your breast implant surgery?	Yes, No, I prefer not to answer
If YES to the last question, please choose all symptoms you have felt	Check all that apply: Breast pain and/or burning sensations, Discomfort in axilla, Changes in breast size, shape, and/or symmetry, Tingling and/or numbness, Hypersensitivity / Rash, Body ache / Joint pain, Muscle pain, Abnormal fatigue, Abdominal discomfort, Anxiety/Depression/Panic Attacks, Cognitive Dysfunction/Memory changes, I prefer not to answer
How long after the surgery did you start experiencing these symptoms?	Immediately, Between 1 month and 6 months, Between 6 months and 1 year, 1-3 years, 4-6 years, 7-10 years, More than 10 years, I prefer not to answer
Have you been diagnosed with any of these autoimmune diseases?	Check all that apply: Breast Cancer, Crohn's Disease, Diabetes Type 1, Fibromyalgia, Hyperthyroidism, Hypothyroidism, Irritable Bowel Syndrome (IBS), Rheumatoid Arthritis, Ulcerative Colitis, Other (Specify), I prefer not to answer
If YES to the last question, when were you diagnosed?	Open-ended
Do you have a family history of connective tissue or autoimmune disease?	Open-ended (Specify family member(s) and disease(s))
Do you have any food or medication allergies?	Open-ended (List allergies)
Have you had your breast implants removed?	Yes, No, I prefer not to answer
If you answered YES to the last question, date of implant removal	Open-ended (Date)
If you answered YES to the last question, did you remove your implants due to any of the symptoms mentioned above?	Yes, No, I prefer not to answer
If you answered YES to the last question, did you continue to experience symptoms after removing the implants?	Yes, No, I prefer not to answer
